# Supplementary material for: Soil desiccation of different microtopographies on a slope in the loess area of northern Shaanxi, China
Source: PLoS One. 2025 Nov 18;20(11):e0336771. doi: 10.1371/journal.pone.0336771 (PMC12626261; doi:10.1371/journal.pone.0336771)
Supplement: Attachment — Submitted filename: REVIEW PONE-D-25-13266.pdf [file pone.0336771.s009.pdf]

**Soil desiccation of different microtopographies on a slope in the loess area of northern Shaanxi, China PONE-D-25-13266**

As part of the peer review process, I would like to offer several critical observations aimed at enhancing the academic and methodological rigor of this manuscript. The topic is relevant and contributes meaningfully to the understanding of hydrology and soil conservation in the Loess Plateau region. However, there are several areas that require clarification or further examination, particularly regarding the consistency of arguments, methodological validity, representativeness of the data, and the strength of evidence supporting the conclusions. The following comments are intended as constructive feedback to help improve the manuscript prior to publication.

The text mentions that vegetation restoration has been carried out since 1998, but then states that vegetation “does not significantly affect deep soil moisture”. This contradicts the subsequent discussion that vegetation is the main cause of desiccation. In addition, there is no mention of vegetation type, age or density in each micro-topography, which is one of the key variables.

I felt the author needed to display a distribution map of the sampling sites or a detailed topographic map showing the spatial position of the micro-topography.

The authors only used one slope site with multiple micro-topography types in one watershed (Hegou). It is not adequately explained whether this site is representative for the entire Loess Plateau. This risks generalization bias. It is also not statistically explained whether the variation between observation points (n=3 per micro-topography) is sufficient to ensure data reliability and validity. The sample size is very limited given the high spatial variability of soil moisture. How do the authors respond to this?

The authors did not confirm inter-annual consistency or significant seasonal influences, even though the survey was conducted before and after the rainy season in 3 different years.

For depths >100 cm, bulk density is equated to the 90-100 cm layer. This assumption tends to be methodologically weak, as bulk density can increase at depth due to soil pressure. This may cause estimation errors in the calculation of soil water storage (SWS).

The authors adopted soil desiccation index (SDI) with universal thresholds (wilting point 4.7% and SSM = 60% of field capacity). However, there is no local validation that this parameter is suitable for all micro-topographic soil types in the study site. This has the potential to create over/underestimation in the classification of drought degree.

The conclusions made by the authors tend to Exceed the Evidence. For example, the claim that soil desiccation is “caused by vegetation distribution patterns” has not been supported by statistical analysis of the relationship between vegetation and the SDI index. No regression or correlation is shown. In addition, the description of “natural vegetation

recovery” in GU as a cause of soil desiccation is speculative, with no quantitative data on actual root biomass or vegetation structure. How do the authors respond to this?

There is no mention of the influence of previous land management, land use history, or potential disturbances (such as human activities, grazing, etc.) that could affect soil moisture or vegetation. I recommend that this is disclosed, albeit briefly.

The overuse soil moisture value in GU is very high (386.36 mm), but there is no evaluation of whether this value is hydrologically realistic under conditions of 478.3 mm annual rainfall. In addition, the 940 cm thickness of the desiccation layer in PL is quite astonishing, but there is no detailed discussion on how the layer formed to such a depth, even though PL is described as an area with high infiltration and low erosion.

I did not find a section that explicitly discusses the limitations of the study in the conclusion, even though many large assumptions were used.
