# Supplementary material for: Soil desiccation of different microtopographies on a slope in the loess area of northern Shaanxi, China
Source: PLoS One. 2025 Nov 18;20(11):e0336771. doi: 10.1371/journal.pone.0336771 (PMC12626261; doi:10.1371/journal.pone.0336771)
Supplement: Attachment — Submitted filename: Manuscript Number PONE-D-25-13266R1.pdf [file pone.0336771.s011.pdf]

Title: Soil desiccation of different microtopographies on a slope in the loess area of northern Shaanxi, China

Manuscript Number PONE-D-25-13266R1

The authors have made significant improvements to the manuscript. However, I would like to offer some additional suggestions to further strengthen the scientific quality and readability of the manuscript:

Explain that the sample size (n=3 per microtopography) has limitations in capturing the spatial variability of soil moisture in the Loess Plateau. Include a statement that the results should be interpreted with caution, and that further research with a broader sampling design is needed.

If data are available, perform a simple regression or correlation to test the relationship between vegetation parameters (biomass, canopy cover) and SDI. If data are not available, explicitly state that the relationship is still hypothetical based on the literature.

Include brief information (e.g., tables or key data summaries) showing that the characteristics of Hegou represent the Loess Plateau in terms of geomorphology, climate, and soil type.

Review whether the values for overuse soil moisture in GU (386 mm) and dry layer PL (940 cm) are consistent with regional hydrological literature. Include a brief discussion to support the validity of these figures.
