# Supplementary material for: Soil desiccation of different microtopographies on a slope in the loess area of northern Shaanxi, China
Source: PLoS One. 2025 Nov 18;20(11):e0336771. doi: 10.1371/journal.pone.0336771 (PMC12626261; doi:10.1371/journal.pone.0336771)
Supplement: Attachment — Submitted filename: Response to Reviewers.docx [file pone.0336771.s010.docx]

Dear Editors and Reviewers:

Thank you very much for your attention and the reviewers’ evaluation and comments on our paper **“Soil desiccation of different microtopographies on a slope in the loess area of northern Shaanxi, China”** (PONE-D-25-13266R1). Those comments are all valuable and extremely helpful for revising and improving our paper, as well as providing significant guidance for our research. We have studied comments carefully and have made correction which we hope meet with approval. We invite a native English scientist of relative research to polish and edit our wording and language, the details of the changes can be found in the manuscript. Besides, our current manuscript followed the journal formatting guidelines of ***PLOS One***. We hope the latest version of the manuscript could meet the journal’s standard. Revised portion are marked in red throughout the revised manuscript. The main corrections in the paper and the Responses to the reviewer’s comments are as flowing:

NOTE: All the Page and Line numbers where revisions were made refer to the Manuscript and Highlight with marked changes (Revised Manuscript (marked up).docx). The Revised Manuscript (clean copy) Version was the same version of the Revised Manuscript (marked up) with cleaned from all the marks.

Soil desiccation of different microtopographies on a slope in the loess area of northern Shaanxi, China PONE-D-25-13266R1

As part of the peer review process, I would like to offer several critical observations aimed at enhancing the academic and methodological rigor of this manuscript. The topic is relevant and contributes meaningfully to the understanding of hydrology and soil conservation in the Loess Plateau region. However, there are several areas that require clarification or further examination, particularly regarding the consistency of arguments, methodological validity, representativeness of the data, and the strength of evidence supporting the conclusions.

Response: Thank you very much for your comments and suggestions.

The following comments are intended as constructive feedback to help improve the manuscript prior to publication.

Response: Thank you very much for your comments and suggestions.

1. The text mentions that vegetation restoration has been carried out since 1998, but then states that vegetation “does not significantly affect deep soil moisture”. This contradicts the subsequent discussion that vegetation is the main cause of desiccation. In addition, there is no mention of vegetation type, age or density in each micro-topography, which is one of the key variables.

Response: Thanks for your comments. In order to control the serious soil erosion on the Loess Plateau, our governmental department implemented the Returning Farmland to Forestry and Grassland Forestry Ecological Project in 1998, and implemented a vegetation restoration program mainly based on artificial afforestation in sloping arable land above 25°, supplemented by natural restoration. In order to compare the differences between natural and artificial restoration, our research team selected the Hegou watershed as a sample site for natural restoration and the Chaigou watershed as a sample site for artificial restoration, and carried out a watershed pairing study, and our study showed that restoring vegetation entirely by nature takes at least 50 years later than artificial restoration. Since 2016, our research program has ended and the local governmental department planted 2-year old oil pine saplings in the Hegou watershed in the same year in order to fulfill the reforestation task of the forestry department for the purpose of rapid vegetation restoration. The sampling time of our study was in 2017, 2018 and 2019, and the focus of our study was also concerned with deep soil moisture, and the effect of planted creosote bush saplings on deep soil moisture was, in this study, negligible, which was the assumption made when our study was conducted. Our group conducted a study on the plant characteristics of different slope microtopographies, as described in the following references.

Reference:

Wang J, Zhu QK, Qin W, Zhang HZ, Yun L, Xie J, Kuang GM. Differentiation of vegetation characteristics on slope micro-topography of fenced watershed in loess area of north Shaanxi province, northwest China. Chinese Journal of Applied Ecology. 2012; 23: 694-700. (In Chinese)

2.I felt the author needed to display a distribution map of the sampling sites or a detailed topographic map showing the spatial position of the micro-topography.

Response: Thank you very much for your comments and suggestions. A map of the distribution of the different microtopographies of our study and a detailed topographic map can be seen in Fig. 1. The different microtopographies are located on the same side of the slope in the Hegou watershed.

3.The authors only used one slope site with multiple micro-topography types in one watershed (Hegou). It is not adequately explained whether this site is representative for the entire Loess Plateau. This risks generalization bias. It is also not statistically explained whether the variation between observation points (n=3 per micro-topography) is sufficient to ensure data reliability and validity. The sample size is very limited given the high spatial variability of soil moisture. How do the authors respond to this?

Response: Thank you very much for your comments and suggestions. The research scope of different microtopography is the slope scale. Our group found 39 watersheds in Wuji County (a representative area of Loess Plateau) by establishing a 10 km grid point method. Through investigation, summarization and analysis, we creatively put forward the concept of slope microtopography: the local topography with a range of 1 m^2^ or more, which has different shapes and sizes due to the action of soil erosion and other effects and the difference of habitat conditions such as soil moisture, soil nutrients and so on. According to the characteristics of microtopography, there are five types of microtopography: shallow gully, cut gully, collapse, slow platform and steep canyon. Based on the scale of our study, together with our soil moisture surveys in 3 years, 2017, 2018 and 2019, and the depth of sampling (10 m), we chose 3 sample sites for each sampling.

4.The authors did not confirm inter-annual consistency or significant seasonal influences, even though the survey was conducted before and aSer the rainy season in 3 different years.

Response: Thank you very much for your comments and suggestions. Our study focuses on the deep soil moisture in different slope microtopographies, and the seasonality of rainfall affects the soil moisture dynamics. According to the results of long-term positional observations by previous authors, the influence of rainfall on soil moisture at the inter-annual or seasonal scales is mainly affected by the infiltration depth of rainfall, and with reference to the existing studies (see the following literature), in the Loess Plateau region, the annual average infiltration depth of rainfall is around 1 In the Loess Plateau region, the average annual infiltration depth of rainfall is about 1 meter, which does not have a large impact on deep soil moisture.

Reference：

Liu, B., Shao, M. Response of soil water dynamics to precipitation years under different vegetation types on the northern Loess Plateau, China. *J. Arid Land* **8**, 47–59 (2016). https://doi.org/10.1007/s40333-015-0088-y.

5.For depths >100 cm, bulk density is equated to the 90-100 cm layer. This assumption tends to be methodologically weak, as bulk density can increase at depth due to soil pressure. This may cause estimation errors in the calculation of soil water storage (SWS).

Response: Thank you very much for your comments and suggestions. Measurement of soil capacity needs to take in situ soil samples, the deeper the soil depth, the more difficult it is to take in situ soil samples, based on the results of the study in the Loess Plateau and the existing reports, the soil capacity in the soil layer 1m range of large variations in the range of 1m, in the range of 1m, the change is not great, we are in the calculation of 1m depth of the soil water storage below, the soil capacity of the soil weight data can be taken in the soil capacity of the approximate depth of the soil layer of 90-100cm. value.

6.The authors adopted soil desiccation index (SDI) with universal thresholds (wilting point 4.7% and SSM = 60% of field capacity). However, there is no local validation that this parameter is suitable for all micro-topographic soil types in the study site. This has the potential to create over/underestimation in the classification of drought degree.

Response: Thank you very much for your comments and suggestions. In our study, when calculating the soil desiccation index (SDI), the wilting humidity of the soil was used as 4.7%, and the stabilized water holding capacity of the soil was used as 60% of the field holding capacity, and the soil type of Wuji County is a yellow mian soil widely developed on loess parent material after stripping of the zonal black clayey soil, with light loamy texture, a field holding capacity of 20.9%, and a wilting humidity of 4.7%. The soil type in our study area, the Hegou watershed, was found to be light loamy in texture and the soil type was also yellow mian soil through preliminary research. Therefore, we took the value of wilting moisture as 4.7% and field water holding capacity as 20.9% for calculating the soil drying flower index.

7.The conclusions made by the authors tend to Exceed the Evidence. For example, the claim that soil desiccation is “caused by vegetation distribution patterns” has not been supported by statistical analysis of the relationship between vegetation and the SDI index. No regression or correlation is shown. In addition, the description of “natural vegetation recovery” in GU as a cause of soil desiccation is speculative, with no quantitative data on actual root biomass or vegetation structure. How do the authors respond to this?

Response: Thank you very much for your comments and suggestions. Our findings are based on the SDI, and we have put forward some speculations in the discussion section that the reason for the occurrence of soil desiccation is mainly due to the influence of vegetation and other factors, based on the existing studies. According to the results of the existing studies in our group, or according to the results of our survey, Table 1 also shows that the plant biomass is larger in GU, which may be the cause of the moderate desiccation in GU, while the ephemeral gully (EG)，the sink hole (SH), the scarp (SC) and the platform (PL) show mild desiccation, which is likely to be caused by climatic drought.

8.There is no mention of the influence of previous land management, land use history, or potential disturbances (such as human activities, grazing, etc.) that could affect soil moisture or vegetation. I recommend that this is disclosed, albeit briefly.

Response: Thank you very much for your comments and suggestions. The effects of prior land management, land use history, or potential disturbances for the different slope microtopographies studied have been added to the text accordingly, as detailed in L101-L105.

9.The overuse soil moisture value in GU is very high (386.36 mm), but there is no evaluation of whether this value is hydrologically realistic under conditions of 478.3 mm annual rainfall. In addition, the 940 cm thickness of the desiccation layer in PL is quite astonishing, but there is no detailed discussion on how the layer formed to such a depth, even though PL is described as an area with high infiltration and low erosion.

Response: Thank you very much for your comments and suggestions. Our conclusions were based on the SDI values according to the delineation criteria. GU had the largest soil moisture overdepletion of 386.36 mm, which was not only consumed by plant growth, but also due to climate change and other effects such as increased evaporation from soil due to higher temperatures in the Loess Plateau in recent years.

Although the soil dry layer thickness of PL reached 940 cm, the degree of soil desiccation was mild and the desiccation index SDI was the lowest, only 12%. This indicates that soil desiccation occurs in PL but is not severe and is also most likely due to climate change. This requires further research.

10.I did not find a section that explicitly discusses the limitations of the study in the conclusion, even though many large assumptions were used.

Response: Thank you very much for your comments and suggestions. Already added in the discussion, see L317-L318.
