# Supplementary material for: Soil desiccation of different microtopographies on a slope in the loess area of northern Shaanxi, China
Source: PLoS One. 2025 Nov 18;20(11):e0336771. doi: 10.1371/journal.pone.0336771 (PMC12626261; doi:10.1371/journal.pone.0336771)
Supplement: Attachment — Submitted filename: Response_to_Reviewers_auresp_2.docx [file pone.0336771.s012.docx]

Dear Editors and Reviewers:

Thank you very much for your attention and the reviewers’ evaluation and comments on our paper **“Soil desiccation of different microtopographies on a slope in the loess area of northern Shaanxi, China”** (PONE-D-25-13266R1). Those comments are all valuable and extremely helpful for revising and improving our paper, as well as providing significant guidance for our research. We have studied comments carefully and have made correction which we hope meet with approval. We invite a native English scientist of relative research to polish and edit our wording and language, the details of the changes can be found in the manuscript. Besides, our current manuscript followed the journal formatting guidelines of ***PLOS One***. We hope the latest version of the manuscript could meet the journal’s standard. Revised portion are marked in red throughout the revised manuscript. The main corrections in the paper and the Responses to the reviewer’s comments are as flowing:

NOTE: All the Page and Line numbers where revisions were made refer to the Manuscript and Highlight with marked changes (Revised Manuscript (marked up).docx). The Revised Manuscript (clean copy) Version was the same version of the Revised Manuscript (marked up) with cleaned from all the marks.

Soil desiccation of different microtopographies on a slope in the loess area of northern Shaanxi, China PONE-D-25-13266R1

As part of the peer review process, I would like to offer several critical observations aimed at enhancing the academic and methodological rigor of this manuscript. The topic is relevant and contributes meaningfully to the understanding of hydrology and soil conservation in the Loess Plateau region. However, there are several areas that require clarification or further examination, particularly regarding the consistency of arguments, methodological validity, representativeness of the data, and the strength of evidence supporting the conclusions.

Reviewer #1: The authors have made significant improvements to the manuscript. However, I would like to offer some additional suggestions to further strengthen the scientific quality and readability of the manuscript:

1. Explain that the sample size (n=3 per microtopography) has limitations in capturing the spatial variability of soil moisture in the Loess Plateau. Include a statement that the results should be interpreted with caution, and that further research with a broader sampling design is needed.

Reponse: Thank you very much for your comments and suggestions. Our sampling method is feasible: one end of the 1m iron rod is connected to the handle with a removable screw, and the other end is connected to the soil sampling soil auger with a removable screw, and then 1m iron rod followed by 1m iron rod is connected to each other in such a way that 10 inter-connected ones will be able to pick up soil samples up to a depth of 10 meters. The exact process is shown in the diagram below.

Figure.1 Soil sampling

On one hand, the collection of soil samples presents certain difficulties. On the other hand, the microtopographies under study vary in scale from 1 m² to 100 m². Therefore, only three sampling points were established for each microtopography. During site selection, we prioritized central locations within each microtopography to avoid edge effects near transitional zones between different microtopographies. The conclusions drawn in this study are preliminary, and further data observation is required for verification.

[1]ZHU Q K, ZHANG Y, ZHAO L L, et al. Vegetation Restoration and Near-Natural Afforestation in the Loess Plateau of Northern Shaanxi[M]. Beijing: Science Press, 2012.

2.If data are available, perform a simple regression or correlation to test the relationship between vegetation parameters (biomass, canopy cover) and SDI. If data are not available, explicitly state that the relationship is still hypothetical based on the literature.

Reponse：Thank you very much for your comments and suggestions. Currently, the available observational data is insufficient for establishing a simple regression or correlation between vegetation parameters (biomass, canopy cover) and SDI. More extensive experimental observations are required. Furthermore, SDI is influenced not only by vegetation factors but also by topographic and climatic factors. The formation of soil dry layers results from the combined effects of human activities and climate change. In analyzing the relationship between vegetation factors and SDI, we based our discussion on the available data. It is proposed that in microtopographies with higher SDI values, the better soil moisture conditions in shallow layers may have facilitated preliminary vegetation recovery. To ensure better survival, vegetation subsequently utilized deeper soil moisture, ultimately leading to moisture deficit in deep soil layers and an increase in SDI.

3.Include brief information (e.g., tables or key data summaries) showing that the characteristics of Hegou represent the Loess Plateau in terms of geomorphology, climate, and soil type.

Reponse：Thank you very much for your comments and suggestions. Hegou is a representative site on the Loess Plateau that our research team has long utilized for eco-hydrological studies. For detailed characteristics of its geomorphology, climate conditions, and soil types, please refer to publications ^[2-3]^ from our research team.

[2] Zhao W J, Zhang Y, Zhu Q K, Qin W, Peng S Z, Li P, Zhao Y M, Ma H, Wang Y. Effects of microtopography on spatial point pattern of forest stands on the semi-arid Loess Plateau, China[J]. Journal of Arid Land, 2015a, 7(3):370-380.

[3]Ma H, Zhu Q K, Zhao W J. Soil water response to precipitation in different micro-topographies on the semi-arid Loess Plateau China[J]. Journal of Forestry Research, 2020, 31(1):245-256.

4.Review whether the values for overuse soil moisture in GU (386 mm) and dry layer PL (940 cm) are consistent with regional hydrological literature. Include a brief discussion to support the validity of these figures.

Reponse：Thank you very much for your comments and suggestions. Previous studies in our research area (Wuqi County) have documented the occurrence of soil desiccation. Observations from depths of 0–9 m indicate that the soil dry layer has extended beyond 9 meters ^[4]^.

[4] Liu G, Wang ZQ, Wang XL. Analysis of dried soil layer of different vegetation types in Wuqi county. Research of Soil and Water Conservation. 2004; 1: 126-129. (In Chinese)

Reviewer #3: The manuscript “Soil desiccation of different microtopographies on a slope in the loess area of northern Shaanxi, China” (PONE-D-25-13266R1) addresses an important and relevant topic for soil hydrology and ecological restoration on the Loess Plateau. The revisions made since the first round improve the clarity of the study design and presentation. However, I find that one of my major concerns from the first review remains insufficiently addressed, and this limits the scientific rigor and broader relevance of the paper.
 In my initial review, I explicitly requested that the discussion should incorporate and critically engage with the latest studies on climate change impacts on soil moisture and desiccation (including but not limited to doi: [10.2166/wcc.2024.207] and [10.1016/j.catena.2021.105633]). These studies provide essential context for linking the observed soil desiccation patterns to broader regional and global climate-change processes.
 In the revised manuscript, the authors briefly mention “climate change” as a contributing factor but do not substantively integrate recent research into their literature review or discussion. This omission weakens the paper, as the findings are not sufficiently situated within the current state of knowledge. Without this, the paper remains largely descriptive and local in scope, rather than demonstrating its broader significance.

Reponse：Thank you very much for your comments and suggestions. The authors have addressed this concern by incorporating discussions on climate change impacts in the designated sections (L316-L323 and L485-L497) of the revised manuscript.
